# Review of "Methods for testing and evaluating survey questionnaires" by S. Presser, et al

**DOI:** 10.1186/1475-925X-4-3

**Published:** 2005-01-06

**Authors:** Kader Parahoo

**Affiliations:** 1 The University of Ulster, Northern Ireland, UK

Questionnaires are, by far, the most common method of data collection in the world. There is hardly anyone who has not been asked to answer questions from one form of questionnaire or another. Familiarity with the method may, on the other hand, give the impression that they are easy to develop and that their findings are unproblematic. For those who construct questionnaires, this is a laborious, time-consuming, artful exercise which requires considerable skills and expertise. In quantitative research, the validity and reliability of the instruments are crucial to the credibility of the findings. The rigorous development and testing of questionnaires are, therefore, essential for producing valid and reliable findings. Yet, as the authors of this book point out, "most text books offer minimal, if any guidance about pretesting methods".

Since the mid-1930's we have learnt a lot about how to make survey instruments, in particular questionnaires, more rigorous and user friendly. Much of this progress was achieved in the last two decades. This book represents this body of work. It reviews key research studies which have evaluated the various techniques and strategies used to enhance the validity and reliability of survey questionnaires.

The idea of this 'monograph' (as the authors call it) was conceived at the Spring 1999 Questionnaire Evaluation Standards International Work Group meeting in London. The chapters evolved out of selected abstracts submitted to the International Conference on Questionnaire Development, Evaluation and Testing Methods in 2002 in South Carolina. As such they represent the latest thinking of an international array of experts on this topic.

The book is divided into the following seven parts: 'cognitive interviews', 'supplements to conventional pretests', 'experiments', 'statistical modelling', 'mode of administration', 'special populations' and 'multimethod applications'. The twenty five chapters go well beyond conventional testing methods and reviews the different ways which can be used to evaluate survey questionnaires. Additionally the book sets the agenda for future research on this topic. It makes a significant contribution to the development and testing of questionnaires.

The book is well sign-posted and the style is clear. I found it both very informative and interesting. It should appeal to those who construct questionnaires for the first time as well as to more experienced researchers. It is likely to become a reference text which research students, at any level, would find it hard to do without.

